# Productivity and Efficiency Growth During Emergency Medicine Residency Training

**DOI:** 10.5811/westjem.21227

**Published:** 2025-02-05

**Authors:** Matthew T. Singh, David M. Austin, Stephanie C. Mullennix, Joshua C. Reynolds, J. Adam Oostema

**Affiliations:** * Corewell Health/Michigan State University College of Human Medicine, Grand Rapids, Michigan; † Emergency Care Specialists, Grand Rapids, Michigan; ‡ Grand Valley State University, Grand Rapids, Michigan

## Abstract

**Introduction:**

Throughout training, an emergency medicine (EM) resident is required to increase efficiency and productivity to ensure safe practice after graduation. Multitasking is one of the 22 Accreditation Council for Graduate Medical Education (ACGME) EM milestones and is often measured through evaluations and observation. Providing quantitative data to both residents and residency administration on patients seen per hour (PPH) and efficiency could improve a resident experience and training in many ways. Our study was designed to analyze various throughput metrics and productivity trends using applied mathematics and a robust dataset. Our goals were to define the curve of resident PPH over time, adjust for relevant confounders, and analyze additional efficiency metrics related to throughput such as door-to-decision time (DTDT).

**Methods:**

We used a retrospective, observational design in a single, tertiary-care center emergency department (ED) that sees approximately 110,000 adult patients per year; our study spanned the period July 1, 2019–December 31, 2021. A total of 42 residents from an ACGME-accredited three-year residency were included in the analysis. We excluded patients <18 years of age. Data was collected using a secure data vendor, and we created an exponential regression model to assess resident PPH data. Additional models were created accounting for patient covariates.

**Results:**

We analyzed a total of 79,232 patients over 30 months. Using an exponential equation and adjusting for patient covariates, median PPH started at 0.898 and ended at 1.425 PPH. The median PPH by postgraduate (PGY) year were 1.13 for PGY 1; 1.38 for PGY 2; and 1.38 for PGY 3. Median DTDT in minutes was as follows: 185 minutes for PGY 1; 171 for PGY 2; and 166 for PGY 3.

**Conclusion:**

Productivity and efficiency metrics such as PPH and DTDT data are an essential part of working in an ED. Our study shows that residents improve with number of patients seen per hour over three years but tend to plateau in their second year. Door-to-decision time continued to improve throughout their three years of training.

Population Health Research Capsule What do we already know about this issue?

*Emergency medicine (EM) residents are expected to grow in both efficiency and productivity during training.*

What was the research question?

*Can a predictive model of EM resident productivity and efficiency growth be defined?*

What was the major finding of the study?

*Patients seen per hour plateaued: PGY 1, 1.13; PGY 2, 1.38, and PGY 3, 1.38. Decision times (in minutes) continued to improve: PGY 1, 185; PGY 2, 171; and PGY 3, 166.*

How does this improve population health?

*Ensuring that EM residents are trained in both efficient and productive patient care is essential to provide safe and effective care.*

## INTRODUCTION

As a specialty dedicated to acute, unscheduled care, the practice of emergency medicine (EM) demands that clinicians not only practice exceptional medicine addressing a wide variety of complaints but do so quickly and efficiently. These skills are important in the setting of increasing patient volumes and illness severity^1^ as well as the downward pressures on EM reimbursement.^2^ In its position statement on emergency department (ED) staffing models, the American College of Emergency Physicians emphasized that determining appropriate and safe staffing models requires an understanding of emergency clinician efficiency.^3^ Efficiency metrics are used not only to ensure adequate staffing but also for emergency physician reimbursement.^4^ Despite the central importance of physician efficiency in clinical practice, EM residency provides little structured education regarding efficiency, and many resident behaviors may affect efficiency.^5^ Moreover, despite the use of robust tools to assess EM resident medical knowledge, clinical reasoning, and interpersonal communication, resident efficiency assessments are largely based on subjective evaluations from supervising physicians.^6^

Although efficiency is an important aspect of practicing EM, current literature suggests that there is wide variability in the measures used to assess clinician efficiency. In a recent scoping review by Anjum et al, patient volume and processing time were two of the most commonly reported metrics to assess productivity and efficiency, respectively.^7^ Providing quantitative resident productivity and efficiency data may help with the clinical growth of residents and help residency administration structure staffing and curricula to better prepare residents for their future practice. Objective measurements of productivity may allow for implementation of interventions and support for residents who are performing below their peers and provide better customized learning experiences for higher performing residents.^8^ It could also help residency clinical competency committees (CCC) to assess resident milestone achievement and ensure graduating residents are ready for independent practice.^9^ Finally, understanding the range and normal trajectory of resident efficiency metrics may be useful to inform staffing model changes in the ED or measure the impact of efficiency-focused curricula.^10^

Previous studies have examined resident efficiency with regard to number of patients seen per hour (PPH) and relative value units; however, many of the studies were performed over a decade ago before the advent of accurate electronic health record (EHR) data.^11^ ^,^ ^12^ There is a lack of data regarding the shape of resident productivity and efficiency growth over the course of training and the effect of patient mix on efficiency. To address this knowledge gap, we used a large administrative dataset to estimate resident productivity and efficiency over the course of training with the goal of defining a curve of resident productivity as well as estimating variability between residents over the course of their training.

## METHODS

### Study Design

In this retrospective observational study we estimated EM resident productivity and efficiency in a cohort of consecutive adult ED patients over the course of 30 months from July 1, 2019–December 31, 2021. The Spectrum Health Institutional Review Board exempted this study as a quality improvement project.

### Study Setting

This study was conducted in a single, regional, tertiary-care center ED, which is a Level I trauma center and comprehensive stroke center. It has an annual volume of approximately 110,000 adult patients per year and regional population of over one million. Patients <18 years of age are not treated in this ED; they are transported to the adjacent children’s hospital unless they require emergent stabilization. We excluded from our analysis any patient <18 years of age in the dataset. Approximately half of the ED footprint is staffed by EM residents, who preferentially see higher acuity, more complex cases with an average admission rate of 42%. The residency program is a three-year training program accredited by the Accreditation Council of Graduate Medical Education. The EM residents work an average of 15 eight-hour shifts per month at this facility. On shift, residents are responsible for direct patient care with attending oversight. Senior residents do not directly supervise more junior residents. As residents progress into postgraduate year (PGY) 2 and PGY 3, they are expected to see higher acuity patients and more complexity. Non-EM residents work on this training site but account for less than 20% of the total residents and were not included in this analysis.

### Data Source and Study Population

We used an administrative dataset that includes all ED visits at the study hospital. This is electronically extracted from the hospital EHR and contains patient-level demographics, limited clinical data, throughput metrics, testing details, disposition, and treating clinicians. We included all adult patients treated by at least one EM resident during an ED visit. We excluded patient encounters for non-EM residents or patients who had no resident contact.

### Exposures and Outcomes

Each patient in the dataset was assigned to the first resident who provided their clinical care. The primary exposure of interest was resident experience as measured by elapsed month of training (1–36). Resident experience was coded at the case level for each encounter by calculating the difference between the calendar month of the visit and the calendar month the resident started residency. Covariates included patient age, sex, Emergency Severity Index (ESI) triage acuity, attending of record, and final disposition (admission vs discharge). The primary productivity outcome was number of patients seen per hour (PPH). Because the administrative dataset did not contain resident shift lengths, we defined shifts by grouping consecutive cases seen by each resident until there was a four-hour gap between registration times. We calculated PPH by dividing this number of cases by the average shift length for residents (eight hours). The primary efficiency outcome was door-to-decision time (DTDT), defined as the time in minutes between ED arrival to disposition decision (placement of an admission or discharge order) as time-stamped in the EHR.

### Statistical Methods

We examined associations between resident month of training and the two primary outcomes using mixed-effects regression models to account for differences in case mix and to quantify the variation in PPH that may be attributable to the individual residents or attendings. In these models, resident experience (in months), patient age, sex, ESI triage acuity, and admission status were treated as patient-level fixed effects while the resident and attending caring for the patient were treated as crossed random effects. This approach was used because residents work with various attendings and vice versa. These models allow for estimation of associations between patient-level characteristics and resident productivity as well as quantifying the contribution of resident- and attending-level variability using the intraclass correlation coefficient (ICC). This statistic may be understood as the proportion of variation in each outcome that is explained by a patient being cared for by an individual resident or supervised by an individual attending. This analysis was then repeated for each postgraduate year of training to examine whether the resident-level variability differed over the course of training. Additionally, to gain some understanding into variability over time, models were repeated in samples limited to each postgraduate year.

Next, using exponential regression we developed figures demonstrating the trajectory of resident productivity (PPH) and efficiency (DTDT) over the course of training. We then developed models using resident experience level as a lone predictor variable as well as models accounting for patient-level covariates (patient age, ESI triage acuity, and admit status). We developed exponential models using Python’s script library (Python Software Foundation, Wilmington, DE) and mixed-effects regression models using Stata version 15 (StataCorp, College Station, TX).

## RESULTS

A total of 79,232 patients encounters that involved a resident were identified over 30 months from July 1, 2019–December 31, 2021. The sample contained 42 distinct residents who worked an estimated 8,378 shifts and accounted for 806 resident-months of training. Characteristics of the patient population and the analyzed residents are presented in Table 1 and Table 2, respectively.

**Table 1.** Characteristics of the patient population.

Patient characteristics	Patient encounters N = 79,232 (%)
Age	
18 to 39	23,400 (29.5)
40 to 59	22,351 (57.7)
60 to 79	23,787 (30.0)
80 or greater	9,694 (12.2)
Female sex	40,617 (51.3)
ESI triage acuity	
Level 1	5,637 (7.2)
Level 2	40,280 (51.3)
Level 3	29,432 (37.5)
Level 4	2,887 (3.7)
Level 5	270 (0.3)
ED disposition	
Admit	29,734 (38.9)
Discharge	

*ED*, emergency department; *ESI*, Emergency Severity Index.

**Table 2.** Characteristics of resident population.

Resident characteristics	Unique residents N = 42 (%)
Female sex	18 (42.9)
Medical degree	
MD	31 (73.8)
DO	10 (23.8)
MBBS	1 (2.4)
Unique resident shifts	8,378
Resident months	806
Median resident PPH	1.4 (1.1–1.6)
PGY-1	1.1 (0.9–1.4)
PGY-2	1.4 (1.3–1.6)
PGY-3	1.4 (1.3–1.8)
Median resident DTDT (minutes)	174 (113–247)
PGY-1	185 (123–254)
PGY-2	171 (119–245)
PGY-3	166 (106–240)

*PGY*, postgraduate year; *PPH*, patients per hour; *DTDT*, door-to-decision time.

### Resident Productivity Over Time

The bivariate associations between the exposures and resident productivity as well as the results of multivariable mixed-effects regression models are presented in Table 3. Patient-level factors associated with reduced PPH included older age, ESI acuity levels 2 and 3 (compared to acuity level 1), and hospital admission. Patient female sex demonstrated no statistically significant association with higher PPH in either unadjusted or adjusted models. Resident experience was positively associated with PPH such that each one month of increased experience was associated with 0.016 additional patients seen per hour (*P* < 0.001). Furthermore, while presence of a supervising attending explained very little of the variability in the number of PPH (ICC = 0.036), resident of record accounted for over 14% of PPH variability (ICC = 0.145). Resident-level ICC statistics changed little across models limited to each postgraduate year (ICC 0.19, 0.23, and 0.15 for PGY 1, 2, and 3). While direct statistical comparisons of these ICCs were not possible, PGY-2 residents demonstrated the numerically greatest between-resident variability.

**Table 3.** Mixed-effects regression models demonstrating associations between patient characteristics and resident productivity as measured by patients seen per hour.

Covariate	Unadjusted coefficients	*P*-value	Adjusted coefficients	*P*-value
Resident experience (per 1 month increase)	0.012 (0.012 to 0.012)	<0.001	0.016 (0.016 to 0.017)	<0.001
Patient age (years)				
18 to 39	Reference		Reference	
40 to 59	−0.035 (−0.043 to −0.028)	<0.001	−0.015 (−0.022 to −0.008)	<0.001
60 to 79	−0.057 (0.065 to −0.049)	<0.001	−0.029 (−0.037 to −0.022)	<0.001
80 or greater	−0.065 (−0.074 to −0.054)	<0.001	−0.029 (−0.039 to −0.02)	<0.001
Patient sex (female vs male)	0.005 (0 to 0.011)	0.08	0.005 (0 to 0.01)	0.07
ESI triage acuity				
Level 1	Reference		Reference	
Level 2	−0.033 (−0.044 to 0.021)	<0.001	−0.024 (−0.035 to −0.013)	<0.001
Level 3	0.003 (−0.009 to 0.148)	0.64	−0.01 (−0.022 to 0.001)	0.08
Level 4	0.140 (0.121 to 0.158)	<0.001	0.093 (0.075 to 0.111)	<0.001
Level 5	0.219 (0.168 to 0.270)	<0.001	0.155 (0.109 to 0.202)	<0.001
Hospital admission (vs discharge)	−0.036 (−0.042 to −0.030)	<0.001	−0.022 (−0.028 to −0.016)	<0.001
Resident ICC			0.145	
Attending ICC			0.036	

*ESI*, Emergency Severity Index; *ICC*, intra-class correlation coefficient.

**Figure 1.**
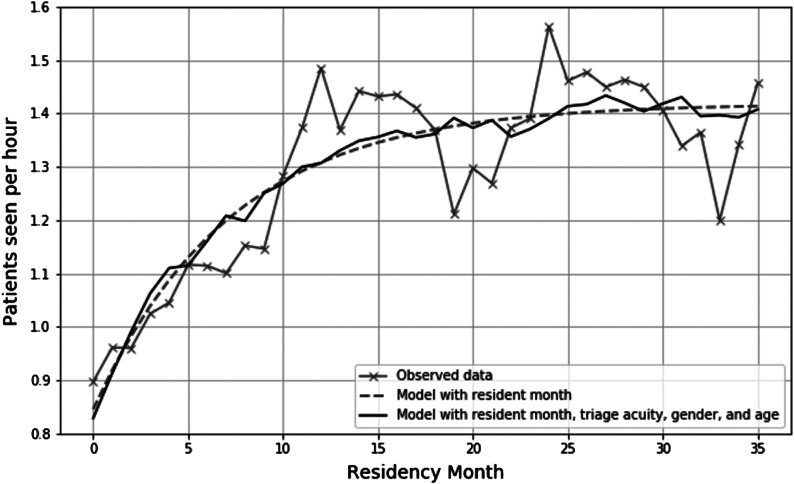
Residency productivity measured by patients seen per hour over the course of training.

Results of the best-fit exponential model of resident productivity over time are presented in Figure 1. Resident productivity increases most rapidly during the first 12 months of residency with little meaningful change beyond the beginning of PGY-2 year. This relationship was consistent even after accounting for patient-level covariates (age, sex, ESI triage acuity).

### Resident Efficiency Over Time

Bivariate associations between the exposure variables and DTDT and the results of multivariable mixed- effects regression models are presented in Table 4. As with models of resident productivity, age >60 years of age was associated with reduced efficiency (longer DTDT), as was ESI triage acuity 2–4 and hospital admission. Female patients had a six-minute longer DTDT compared to males (*P* < 0.001). When examining group-level contributions to variability in DTDT, neither attending (ICC = 0.008) nor resident (ICC = 0.012) accounted for a meaningful proportion of observed variability.

**Table 4.** Mixed-effects regression models demonstrating associations between patient characteristics and resident efficiency as measured by door-to-decision time.

Covariate	Unadjusted coefficients	*P*-value	Adjusted coefficients	*P*-value
Resident experience (per 1-month increase)	−0.91 (−1.01 to −0.82)	<0.001	−0.44 (−0.56 to −0.31)	<0.001
Patient age (years)				
18 to 39	Reference		Reference	
40 to 59	12.33 (9.86 to 14.80)	<0.001	10.88 (8.52 to 13.24)	<0.001
60 to 79	10.33 (7.91 to 12.74)	<0.001	9.30 (6.91 to 11.69)	<0.001
≥80	9.89 (6.76 to 13.02)	<0.001	9.06 (5.98 to 12.14)	<0.001
Patient sex (female vs male)	6.37 (4.51 to 8.23)		6.51 (4.74 to 8.27)	<0.001
ESI triage acuity				
Level 1	Reference		Reference	
Level 2	103.06 (99.52 to 106.59)	<0.001	101.11 (97.61 to 104.60)	<0.001
Level 3	84.38 (80.75 to 88.00)	<0.001	87.74 (84.00 to 91.48)	<0.001
Level 4	12.09 (6.15 to 18.02)	<0.001	20.89 (14.91 to 26.87)	<0.001
Level 5	−8.09 (−24.55 to 8.37)	0.36	0.66 (−15.31 to 16.62)	0.94
Hospital admission (vs discharge)	−0.86 (−2.69 to 0.97)	0.36	4.26 (2.25 to 6.28)	<0.001
Resident ICC			0.012	
Attending ICC			0.008	

*ESI*, Emergency Severity Index; *ICC*, intra-class correlation coefficient.

**Figure 2.**
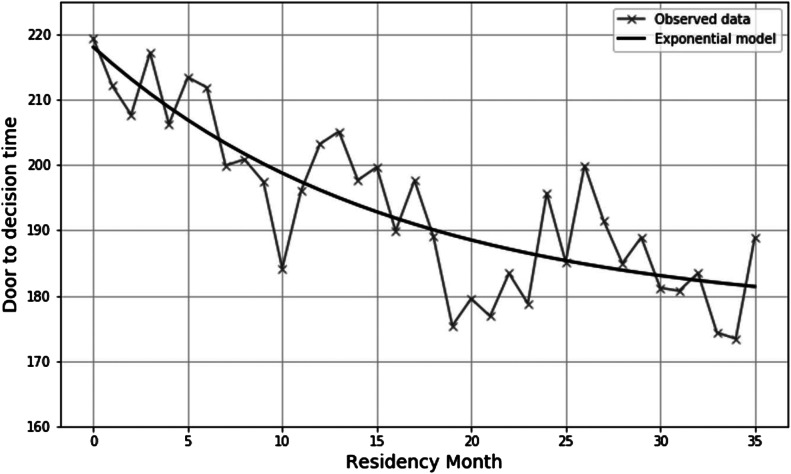
Residency productivity measured by door-to-decision time over the course of training.

Results for exponential models of resident efficiency over time are presented in Figure 2. The rate of change observed in DTDT was less than and more gradual than the number of PPH over the course of residency training, with improvement levelling off during the PGY-3 year.

## DISCUSSION

Throughout training, EM residents must develop skills in managing the complex needs of multiple patients such that they become both productive and efficient. These skills are undoubtedly important and are logical elements to evaluate over the course of residency training. Nevertheless, few existing competencies address these aspects of practice directly, and their assessment is primarily dependent upon subjective evaluation by attending physicians. In this analysis we sought to quantify resident productivity and efficiency in our institution over the course of residency training through examining the number of PPH and DTDT.

We observed that resident productivity increases dramatically during the first year of residency but levels off early in the PGY-2 year, similar to plateaus described in previous studies.^12^ ^–^ ^14^ Patient-level factors such as older-age patients requiring hospital admission were associated with lower PPH, while patients triaged as lower ESI acuity (especially levels 4 and 5) were associated with higher PPH similar to attending-based studies of PPH by Joseph et al.^15^ After accounting for these relationships, the independent association between experience and productivity was actually stronger, suggesting that residents become more efficient over the course of training while also seeing a more difficult case mix.

We also observed significant between-resident variability. Overall, individual residents accounted for over 14% of observed variation in PPH after accounting for patient-level factors, while attending physicians contributed very little (3.6%). Furthermore, the degree of variability attributable to individual residents after accounting for case mix was highest for PGY-2 residents, but lower among PGY-1 and PGY-3 residents. This may suggest that residents begin and end their training at similar levels of productivity but may reach their plateau at different points during PGY 2. These findings suggest that productivity is in part an attribute of individual residents rather than case mix or attending staffing practices. Thus, there may be a role for designing education interventions for residents who demonstrate lower productivity by the end of PGY 1. While productivity appears to be an attribute of residents themselves, the improvement in ICC may suggest that residency training does help to reduce performance variability to some degree.

To examine resident efficiency, we chose to evaluate DTDT rather than other throughput makers such as ED length of stay, since DTDT is more likely to reflect resident behavior rather than system factors such as staffing and ED boarding. In contrast to PPH, this metric did not plateau early in residency but rather continued to improve over the course of a resident’s training. As with PPH, patient-level factors were significantly associated with longer DTDT, including older age, ESI triage acuity 2 and 3, and admission status. Relative to other patient-level factors, resident experience level had a more modest relationship with DTDT such that a resident’s experience alone accounted for an approximate 15-minute reduction over the 36 months of training. Furthermore, in contrast to PPH, only 1% of the unexplained variation in DTDT was attributable to the resident providing care (ICC = 0.012), and almost none of it was attributable to the supervising attending (ICC = 0.008). This may be partially explained by the fact that PPH is a metric that is inherently tied to residents, resulting in greater between-resident variation. Nevertheless, it is also likely true that structural limitations (eg, time to lab results, time to consultant phone call return) result in greater homogeny in completing ED workups that may explain this lower level of between-resident variability with regard to DTDT.

Using this data from this analysis raises numerous opportunities for residency administration and assisting residents in maximizing their performance and growth. Recognizing that there is a plateau in the number of PPH during training could help with staffing models to ensure appropriate resident coverage and manage expectations. One potential opportunity to leverage data might be for residency programs to monitor their productivity data several times during an academic year to ensure that their residents are progressing appropriately along the described curve. Residents who are not showing appropriate growth by the end of PGY 1 may benefit from tailored interventions or individualized learning plans. Another consideration relevant to both productivity and efficiency may be to provide residents with their own performance on these metrics in relation to their peers. In our institution, our CCC reviews resident productivity data such as PPH and DTDT twice per year for each residency class. This information is deidentified and distributed to each resident to review with their faculty advisor and program director. This information is frequently used to develop individualized learning plans to help with their patient efficiency and often used to advance their ACGME patient care-related milestones.

## LIMITATIONS

Given that this was an observational analysis, it is important to acknowledge that these models do not prove a causal relationship between any of the potential predictors of PPH or DTDT and their respective outcomes. While PPH and DTDT are recognized benchmarks in many EDs, it is also important to remember that there are other ways to define productivity and efficiency, and several potential confounders may exist in this dataset.^16^ More advanced residents may use their time in other ways such as peer-to-peer teaching, managing a larger volume of “signed-out” patients, more efficiency with on-shift documentation, and less time spent continuing patient care after their shift has ended. These possibilities may not tie directly to patient productivity but may provide value for both the residency and the individualized learner. It is certainly possible that the types of patients cared for by senior residents tended to be more complex even after accounting for ESI triage acuity, resulting in residual confounding. Further studies could evaluate some of these variables to account for why resident efficiency tends to plateau with number of PPH in their second year of residency.

Additionally, while our method of reconstructing shifts based on patient registration times likely results in a reasonably accurate summary, it remains possible that some patients were incorrectly assigned to a shift resulting in under- or overestimation of resident productivity. Finally, our institution diverts lower acuity patients to a “fast-track” area that is not staffed by residents. This likely accounts for the distribution of ESI triage acuity and admission rates, which are higher than a general ED population. Thus, the absolute productivity numbers should be interpreted with caution, and it is difficult to know how these results may compare across institutions.

Another important confounder that may reduce the generalizability of this analysis is the impact of the Covid-19 pandemic. Given that our dataset includes periods impacted by COVID-19, it is possible that this may have influenced resident productivity and efficiency.^17^ There are mitigating factors that suggest our analysis was not adversely affected by the pandemic. First, since our analysis is defined by month of experience rather than calendar time, the impact of COVID-19 was spread equally among training months. Second, due to the module structure of our department, the teaching module is preserved for higher-acuity complaints and is less impacted by low ED volumes or boarding patients than other locations in the ED. We examined overall resident patients and PPH over the calendar duration of the study period and found no meaningful relationship between COVID-19 and non-COVID-19 periods (supplemental figure). Finally, residents were not restricted from managing patients under the investigation of COVID-19.

Lastly, it must be emphasized that productivity and efficiency, while important skills to the emergency clinician, should not supplant or overshadow the many other critical skills that require attention in residency such as acquisition of medical knowledge, effective communication, and the delivery of compassionate, empathetic, and equitable patient care.

## CONCLUSION

This analysis confirms that resident productivity and efficiency improve over the course of residency training. Similar to the findings of previous research, productivity as measured by number of patients seen per hour appears to advance more quickly and reach a plateau by the PGY-2 year. However, efficiency as measured by door-to-decision time improves over the course of training. These relationships persist following adjustment for potential patient-level confounders and, in the case of PPH, are associated with individual residents. Interestingly, attending variability has little effect on PPH. These findings suggest that assessment of these metrics periodically during residency may be helpful in tailoring educational interventions to assist residents in developing these skills. Further study is needed to verify these findings and determine the impact of interventions designed to modify resident productivity and efficiency during training.

## Supplementary Information




