# Supplementary material for: Productivity and Efficiency Growth During Emergency Medicine Residency Training
Source: West J Emerg Med. 2025 Feb 5;26(2):246–53. doi: 10.5811/westjem.21227 (PMC11931712; doi:10.5811/westjem.21227)
Supplement: Supplementary file 1 [file wjem-26-246-s001.docx]

Supplemental Figure: PPH of residents during study throughout Covid-19 Pandemic
